# Eligibility, uptake and response to germline genetic testing in women with DCIS

**DOI:** 10.3389/fonc.2022.918757

**Published:** 2022-08-26

**Authors:** Lauren Turza, Leann A. Lovejoy, Clesson E. Turner, Craig D. Shriver, Rachel E. Ellsworth

**Affiliations:** ^1^ Department of Surgery, Rebecca Fortney Breast Center, Anne Arundel Medical Center, Annapolis, MD, United States; ^2^ Clinical Breast Care Project, Chan Soon-Shiong Institute of Molecular Medicine at Windber, Windber, PA, United States; ^3^ Murtha Cancer Center Research Program, Department of Surgery, Uniformed Services University of the Health Sciences, Bethesda, MD, United States; ^4^ National Human Genome Research Institute, National Institutes of Health, Bethesda, MD, United States; ^5^ Henry M. Jackson Foundation for the Advancement of Military Medicine, Bethesda, MD, United States

**Keywords:** ductal carcinoma *in situ*, genetic testing, germline mutation, risk-reducing surgery, recurrence

## Abstract

**Background:**

Ductal carcinoma *in situ* (DCIS) is a malignant, yet pre-invasive disease of the breast. While the majority of DCIS have low risk of recurrence, a subset of women with germline pathogenic variants (PV) in cancer predisposition genes are at increased risk for recurrence. Uptake of genetic testing and subsequent surgical intervention in women with DCIS has not been well-studied. The aim of this study was to evaluate test eligibility parameters, uptake of clinical testing, impact on surgical decision making and second cancer events (SCE) in women with DCIS.

**Methods:**

Four-hundred eighty-four women diagnosed with unilateral DCIS 2001-2020 were eligible for this study. Demographic, commercial genetic test results and surgical procedures were extracted from the database. Test-eligibility was assigned using National Comprehensive Cancer Network (NCCN) criteria. Panel genetic testing was performed in the research laboratory across 94 cancer predisposition genes. Statistical analyses were performed using Fisher’s exact tests and Chi-square analyses with *p* < 0.05 defining significance.

**Results:**

Forty-four percent of women were test-eligible at diagnosis of which 63.4% pursued genetic testing before definitive surgery; 9.9% pursued testing only after a second cancer event. Bilateral mastectomy (BM) was significantly higher (p<0.001) in women who had testing before definitive surgery (46.9%) compared to those who had testing afterword (10.8%) and in women who underwent testing before definitive surgery with PV (75%) compared to those without PV (37.5%. p=0.045). Of the 39 women with PV, 20 (51.3%) were detected only in the research setting, with 7 (17.9%) of these women not eligible for genetic testing based on NCCN criteria. In women who did not undergo BM at diagnosis, SCE were significantly higher (p=0.001) in women with PV (33.3%) compared to those without PV (11.9%).

**Conclusion:**

Pursuit of genetic testing and subsequent use of risk-reducing surgeries in women with PV was suboptimal in women with a primary diagnosis of DCIS. In conjunction, >50% of PV were detected only in the research setting. Because omission of genetic testing in women with DCIS may represent a lost opportunity for prevention, genetic testing at the time of diagnosis should be standard for all women with DCIS.

## Introduction

Ductal carcinoma *in situ* (DCIS) is a disease of the breast in which epithelial cellular proliferation fills terminal ductal lobular units with malignant cells. Although a pre-invasive condition, DCIS is a non-obligate precursor to invasive breast cancer (IBC) (1). Studies of the natural progression of DCIS found that 14-53% of untreated lesions progressed to IBC (2). Current treatment options for DCIS include surgery, radiation and endocrine therapy (1).

The primary goal of treatment of DCIS is to prevent recurrence (3). To avoid over- or under-treating indolent or aggressive DCIS, a number of studies and clinical trials are attempting to identify pathological characteristics and biomarkers associated with risk of recurrence and evaluating whether active surveillance is an acceptable alternative to surgery for some women (4). One subset of women with DCIS who may benefit not only from more extensive breast surgical options including bilateral mastectomy (BM), but bilateral salpingo-oophorectomy as well, are those who harbor pathogenic variants (PV) in cancer predisposition genes associated with increased risk for recurrence and secondary cancers at other sites.

In the United States, guidelines, such as those issued by the National Comprehensive Cancer Network (NCCN), are used to identify those women with breast cancer, both invasive and DCIS, who are likely to benefit from germline genetic testing. Recent studies suggest that a significant number of women with PV do not meet testing criteria (5–7) and thus miss the opportunity to pursue risk-reducing strategies, including BM, at diagnosis. In response, the American Society of Breast Surgeons (ASBS) recommends genetic testing should be offered to all women with breast cancer (8). This study was designed to evaluate how effective current test eligibility parameters are in identifying women with germline mutations in cancer predisposition genes, uptake and timing of genetic testing, choice of risk-reducing surgery in those with PV and second cancer events (SCE) in a cohort of 484 women with a primary diagnosis of unilateral DCIS.

## Materials and methods

### Patient eligibility and enrollment

All subjects in this study voluntarily agreed to participate in the Clinical Breast Care Project (CBCP). Patients were enrolled and treated at the CBCP member sites of Walter Reed National Military Medical Center (WRNMMC), Bethesda, MD (n=266), Anne Arundel Medical Center, Annapolis, MD (n=159) or Joyce Murtha Breast Care Center, Windber, PA (n=59). Demographic and clinical data and blood samples were collected with approval from the WRNMMC Human Use Committee and Institutional Review Board.

### Patient data

Demographic, personal and family health history and lifestyle factors were collected and entered into the CBCP database at the time of diagnosis. Clinical data such as pathological characteristics and germline test results were entered into the CBCP database as they became available. Follow-up data, including any additional cancer events, was collected annually and entered into the CBCP database. Eligibility for germline testing was assigned using National Comprehensive Cancer Network (NCCN) criteria, which includes age at diagnosis and personal and family cancer history, both from the year of diagnosis and criteria from 2021 (version 2.2021). Type of surgical management, local, regional and distant SCE and patient status through December 31, 2021 were collected. Results from clinical genetic testing (n=110) were extracted from the CBCP database.

### Data generation and analysis

Genomic DNA was available from 465 women, 92 of whom also had clinical genetic testing performed. Genomic DNA was isolated from blood samples as previously described (9). Sequencing libraries were created using Illumina DNA prep with enrichment kits and the TruSight Cancer panel and sequenced on a MiSeq (Illumina, Inc, San Diego, CA) according to manufacturer’s protocols. Data were analyzed and variants classified as previously described (9). Statistical analyses were performed using Fisher’s exact tests and Chi-square analyses. Statistical significance was defined as *p* < 0.05.

## Results

### Cohort characteristics

Four hundred eighty-four women with a primary diagnosis of unilateral DCIS were eligible for this study. The average age at diagnosis in this cohort was 57 years. Average follow-up time was 8.6 years. Fifty-nine (12.2%) women had second cancer events including 13 recurrences (6 ipsilateral DCIS, 7 ipsilateral IBC) and 43 second cancer events (7 DCIS, 36 IBC), ovarian cancer (n=1) and metastatic spread without detection of IBC (n=2). One woman who developed ipsilateral invasive breast cancer died of disease.

### Test eligibility

**Figure 1:**
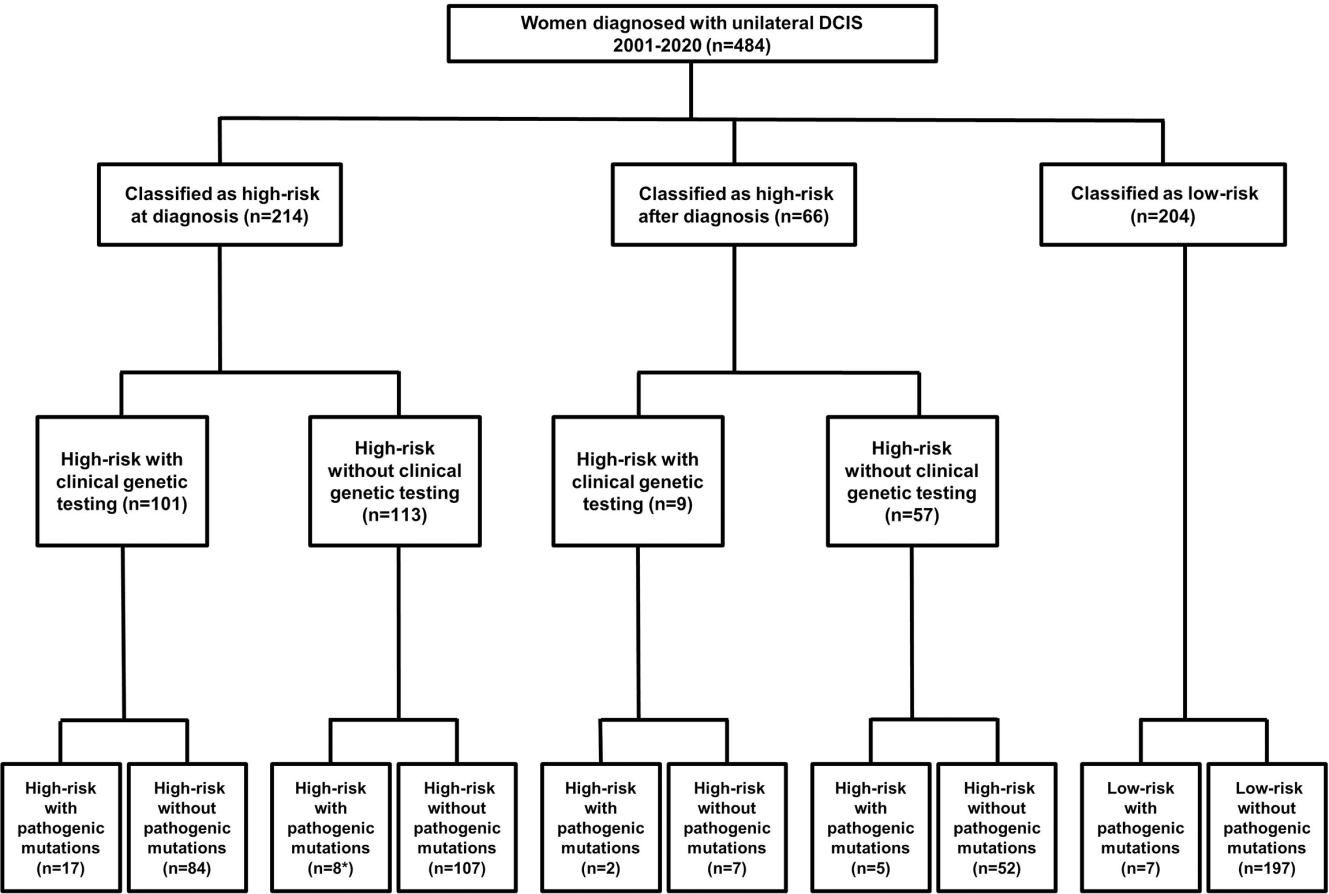
Flow-chart detailing patient risk, test uptake and detection of pathogenic mutations. *Two women who had clinical testing limited to *BRCA1* and *BRCA2* with no PV reported had PV in the *ATM* and *CHEK2* genes detected in the research setting.

Two-hundred fourteen women met at least one NCCN criteria for genetic testing at the time of diagnosis (**Figure 1**). An additional 66 women met test criteria from 2021, including 21 women whose test status changed only after subsequent diagnosis of recurrent DCIS (n=3), IBC (n=17) or ovarian cancer (n=1). There was no significant difference in patient self-reported race/ethnicity between risk-groups, however, women who were test-eligible at the time of diagnosis had more relatives with breast, ovarian or pancreatic than those with delayed test-eligibility and second cancer events were lowest in low-risk patients (**Table 1**).

**Table 1:** Demographic and clinical information for all patients classified as high-risk at the time of diagnosis, high-risk after diagnosis or low-risk using NCCN criteria.

	High-risk at diagnosis (n=214)	High-risk after diagnosis (n=66)	p-value^a^	Low-risk (n=204)	p-value^b^
Age at Diagnosis	52.3 years	53.0 years	0.680	62.8 years	<0.001
	N (%)	N (%)		N (%)	
Ethnicity			0.879		0.417
Non-Hispanic Black	46 (21.5%)	12 (18.2%)		34 (16.7%)	
Asian/Pacific Islander	7 (3.3%)	3 (4.5%)		12 (5.9%)	
Hispanic	7 (3.3%)	2 (3.0%)		6 (2.9%)	
Non-Hispanic White	152 (71.0%)	49 (74.3%)		148 (72.5%)	
Other/Unknown	2 (0.9%)	0 (0.0%)		4 (2.0%)	
Family history^c^			<0.001		<0.001
0	41^d^ (19.2%)	16 (24.2%)		139 (68.1%)	
1	53 (24.9%)	46 (69.7%)		65 (31.9%)	
2	75 (35.2%)	3 (4.6%)		0 (0.0%)	
>3	44 (20.7%)	1 (1.5%)		0 (0.0%)	
Disease-recurrence			<0.001		<0.001
Yes	28 (13.1%)	21 (31.8%)		10 (4.9%)	
No	186 (86.9%)	45 (68.2%)		194 (95.1%)	

a p-value for women who were test eligible at compared to after diagnosis.

b p-value for high-risk compared to low-risk women.

c Family history of breast, ovarian and pancreatic cancer through third degree family members.

### Test uptake

Overall, 39.3% (110/280) of test eligible women underwent clinical genetic testing. Test uptake was significantly higher (p<0.001) in women who were eligible at the time of testing (47.2%) compared to those with delayed eligibility (13.6%). In women who were test-eligible at the time of diagnosis, those that underwent genetic testing were significantly younger than those who did not pursue genetic testing and were more likely to have ≥3 family members with a history of cancer (**Table 2**).

**Table 2:** Demographic and clinical information for patients classified as test eligible at the time of diagnosis who did or did not pursue genetic testing.

	High-risk tested (n=101)	High-risk not tested (n=113)	P-value
Age at Diagnosis	49.1 years	55.1 years	0.002
	N (%)	N (%)	
Ethnicity			0.455
Non-Hispanic Black	25 (24.7%)	21 (18.6%)	
Asian/Pacific Islander	4 (4.0%)	3 (2.7%)	
Hispanic	4 (4.0%)	3 (2.7%)	
Non-Hispanic White	68 (67.3%)	84 (74.3%)	
Other/Unknown	0 (0.0%)	2 (1.7%)	
Education			0.146
<college degree	38 (37.6%)	55 (48.7%)	
≥college degree	59 (58.4%)	51 (45.1%)	
Unknown	4 (4.0%)	7 (6.2%)	
Family History			0.028
0	22 (21.8%)	20 (17.7%)	
1	24 (23.8%)	29 (25.7%)	
2	27 (26.7%)	48 (42.5%)	
≥3	28 (27.7%)	16 (14.1%)	

### Timing of test uptake

Timing of genetic testing varied. Time-to-testing was significantly (p<0.001) longer in women who became eligible after diagnosis (average 8.94 years, range 3.1 -14.9 years) than those who were eligible at the time of diagnosis (average 2.15 years, range 0-18.5 years). Within the test-eligible at diagnosis cohort of women, 63.4% (64/101) pursued genetic testing before definitive surgery. Ten (9.9%) of the women eligible for testing at diagnosis delayed testing until after a second cancer event. Each of the nine women with delayed eligibility for testing pursued clinical genetic testing after a second cancer event.

### Overall mutation rates

The mutation frequency of women with clinical test results was 17.3% (19/110). Two women who had clinical testing limited to *BRCA1* and *BRCA2*, had PV in *ATM* (n=1) and *CHEK2* (n=1) detected in the research setting. Eleven (6.5%) of an additional 170 test-eligible women who did not pursue clinical testing had PV detected in the research laboratory. Within the test-ineligible population, 7/204 (3.4%) women had PV. In total, 20/39 (51.3%) PV were detected in women who did not undergo clinical genetic testing. PV were detected in 13 cancer predisposition genes including *ATM* (n=4), *BLM* (n=1), *BRCA1* (n=1), *BRCA2* (n=7), *BRIP1* (n=1), *CDKN2A* (n=1), *CHEK2* (n=9), *FANCC* (n=1), *MUTYH* (n=10), *NBN* (n=1), *PALB2* (n=1), *RAD51D* (n=1) and *STK11* (n=1) (**Table 3**). *CHEK2* (n=9) and *BRCA2* (n=7) had the highest frequency of PV in high-risk women while *MUTYH* had the most PV in low-risk women.

**Table 3:** Variants classified as pathogenic or likely pathogenic according to ACMG classification.

Patient	Pathogenic variant	Testing before definitive surgery	Surgery at diagnosis^a^	Recurrence
**Test-eligible with clinical testing**				
17	BRCA2: exact mutation from clinical lab not provided	√	BM/BSO	
48^b^	NM_007194.4(CHEK2):c.470T>C (p.Ile157Thr)		UM	Contralateral IBC^c^
68	NM_007194.4(CHEK2):c.906delA (p.Glu302fs)		BM	
156	NM_000077.4(CDKN2A):c.301G>T (p.Gly101Trp)		BCS	
188	NM_024675.4(PALB2):c.509_510delGA (p.Arg170fs)	√	BM/BSO	
190	NM_001048174.2(MUTYH):c.1103G>A (p.Gly368Asp)		BCS	
364	NM_012222.2(MUTYH):c.724C>T (p.Arg242Cys)		BCS	
369	NM_007194.4(CHEK2):c.85C>T (p.Gln29Ter)		BCS/BSO	
379	NM_000059.4(BRCA2):c.518delG (p.Gly173fs)	√	BM/BSO	
416	NM_000059.4(BRCA2):c.3975_3978dupTGCT (p.Ala1327fs)	√	BCS	Ipsilateral IBC
429	NM_000059.3(BRCA2):c.8902_8913delACCGTGTGGAAinsTCCC (p.Thr2968fs)	√	BM/BSO	
431	NM_000059.4(BRCA2):c.5946delT (p.Ser1982fs)	√	BCS/BSO	Ipsilateral IBC
445	NM_032043.2(BRIP1):c.1045G>C (p.Ala349Pro)		BCS	Ipsilateral IBC
468	STK11: partial gene deletion	√	BM	
476	CHEK2: deletion exons 9-10	√	BM	
504 ^b^	NM_000051.4(ATM):c.6706G>T (p.Glu2236*)		BCS	Ipsilateral IBC
505	NM_000059.4(BRCA2):c.1310_1313del (p.Lys437fs)		BCS	Ipsilateral IBC and OC^d^
518	NM_001048174.2(MUTYH):c.1103G>A (p.Gly368Asp)		BCS	Ipsilateral DCIS
559	NM_007194.4(CHEK2):c.470T>C (p.Ile157Thr)		BCS	
**Test-eligible with research results**				
82	NM_000057.4(BLM):c.1933C>T (p.Gln645*)		BCS	
97	NM_000059.3(BRCA2):c.2842dupG (p.Val948fs)		UM	Contralateral IBC
120	NM_002485.5(NBN):c.698_701del (p.Lys233fs)		BM	
143^e^	NM_000051.4(ATM):c.6228del (p.Leu2077fs)		BCS	
230	NM_002878.3(RAD51D):c.694C>T (p.Arg232*)		BM	
240^e^	NM_007194.4(CHEK2):c.1100delC (p.Thr367fs)		BCS	Ipsilateral DCIS
276	NM_007194.4(CHEK2):c.1100delC (p.Thr367fs)		BCS	
293	NM_007194.4(CHEK2):c.349A>G (p.Arg117Gly)		BCS	
297	NM_000136.3(FANCC):c.355_360delinsA (p.Ser119fs)		UM	
319	NM_007194.4(CHEK2):c.1100delC (p.Thr367fs)		BCS	
405	NM_001048174.2(MUTYH):c.1351G>T (p.Glu451Ter)		BCS	Ipsilateral IBC
427	NM_012222.2(MUTYH):c.724C>T (p.Arg242Cys)		BCS	
511	NM_007294.4(BRCA1):c.4035delA (p.Glu1346fs)		BCS	
**Test-ineligible with research results**				
181	NM_001048174.2(MUTYH):c.452A>G (p.Tyr151Cys)		BCS	
231	NM_000051.3(ATM):c.7096G>T (p.Glu2366*)		BCS	
237	NM_001048174.2(MUTYH):c.452A>G (p.Tyr151Cys)		BCS	
339	NM_000051.4(ATM):c.1564_1565delGA (p.Glu522fs)		BCS	
388	NM_001048174.2(MUTYH):c.1103G>A (p.Gly368Asp)		BCS	
423	NM_001048174.2(MUTYH):c.1103G>A (p.Gly368Asp)		UM	
469	NM_001048174.2(MUTYH):c.1103G>A (p.Gly368Asp)		BCS	

a BCS, breast conserving surgery; UM, unilateral mastectomy; BM, bilateral mastectomy; BSO, bilateral salpingo oophorectomy.

b Patients became eligible for testing only after development of a second breast tumor.

c IBC, invasive breast cancer.

d OC, ovarian cancer.

e Patients had genetic testing of BRCA1 and BRCA2 done clinically; ATM and CHEK2 mutation detected in the research setting.

### Surgical choices and outcomes

Within the 101 women who underwent clinical genetic testing, BM was significantly higher (p<0.001) in women who had testing before definitive surgery (46.9%) compared to those who had testing after definitive surgery (10.8%). Within the women with clinically-detected PV, 36.8% elected for BM. In women who underwent testing before definitive surgery, BM was significantly higher (p=0.045) in those with PV (75%) compared to those without (42.9%). In women who received negative test results before definitive surgery, the rate of SCE was 0% in women who underwent BM and 6% in those who did not (p=0.212). The number of PV in high- and low-risk women who had SCE is shown in **Table 4**. In women who did not undergo BM at the time of diagnosis, SCE were significantly higher (p=0.001) in women with PV (10/30; 33.3%) compared to those women without PV (46/388, 11.9%). None of the women with PV died of disease.

**Table 4:** Risk-status and germline variants in 59 women with second cancer events.

	Ipsilateral DCIS	Contralateral DCIS	Ipsilateral Invasive	Contralateral Invasive	Ovary	Metastatic with no IBC^a^
High-Risk^b^						
High-penetrance	0 (0%)	0 (0%)	^c^3 (12.5%)	1 (8.3%)	0 (0%)	0 (0%)
Moderate-penetrance	^d^1 (14.3%)	0 (0%)	2 (8.3%)	1 (8.3%)	0 (0%)	0 (0%)
Other	^e^1 (14.3%)	0 (0%)	1 (4.2%)	0 (0%)	0 (0%)	0 (0%)
No PV	^f^5 (71.4%)	3 (100%)	^h^18 (75.0%)	10 (83.3%)	1 (100%)	2 (100%)
Low-Risk						
High-penetrance	0 (0%)	0 (0%)	0 (0%)	0 (0%)	0 (0%)	0 (0%)
Moderate-penetrance	0 (0%)	0 (0%)	0 (0%)	0 (0%)	0 (0%)	0 (0%)
Other	0 (0%)	0 (0%)	0 (0%)	0 (0%)	0 (0%)	0 (0%)
No PV	2^g^ (100%)	1 (100%)	3 ^i^ (100%)	4 (100%)	0 (0%)	0 (0%)

a IBC, invasive breast cancer.

b High-penetrance genes: BRCA2, moderate penetrance genes: ATM, BRIP1, CHEK2.

c 1 of 3 patients had recurrent disease.

d This patient had recurrent disease.

e This patient had recurrent disease.

f 2 of 5 patients had recurrent disease.

g 5 of 18 patients had recurrent disease.

h 1 of 2 patients had recurrent disease.

i 2 of 3 patients had recurrent disease.

## Discussion

The primary goal of treatment for DCIS is prevention of IBC. Although a significant number of women have indolent forms of DCIS and may be effectively treated using active surveillance rather than surgical interventions (4), those with hereditary forms of DCIS are at increased risk for additional breast cancers, both ipsilateral and contralateral, as well as secondary tumors in other organ sites. For these patients, surgical management may be more extensive and include BM for women with PV in *BRCA1*, *BRCA2*, *PALB2* and *TP53*, and BSO for women with PV in *BRCA1*, *BRCA2, BRIP1*, *RAD51C* and *RAD51D* (10, 11). Identification of women with heritable forms of DCIS is, therefore, critical in surgical decision making and preventing disease recurrence.

Efficacy of germline testing in reducing the risk of additional cancers in patients with DCIS is dependent on several factors, including test-eligibility, timing of genetic testing and treatment decisions based on underlying PV. In this study, 44.2% of women were eligible for testing at diagnosis; this was not significantly different (p=0.944) from a cohort of women with IBC (44.0%) diagnosed over the same time period (9); test uptake was <50% in test-eligible at diagnosis women with DCIS (47.2%) but not significantly lower (p=0.241) than those with IBC (51.8%). In our study, 40.6% (13 of 32) of PV in test-eligible women were not detected clinically. In addition, the frequency of PV in test-ineligible women was 3.4% in women with DCIS, similar to the 4.0% detected in women with invasive breast cancer (9). These data suggest that the use of test-eligibility criteria may create a failed opportunity for prevention; implementation of the the ASBS recommendation that all women with breast cancer should be offered genetic testing (8) may reduce risk of recurrence in women with DCIS.

The timing of genetic testing also is also important as it can lead to changes in surgical decision making. Of the 101 women who were test-eligible at the time of diagnosis with clinical test results, 36 (35.6%) underwent genetic testing after definitive surgery for DCIS, with 10 (9.9%) delaying testing until after a second cancer event. Ten (27.0%) women with delayed testing were found to harbor PV, six of whom had PV in genes for which NCCN recommendations for patient management are available.

Genetic testing results can reduce future cancer risk when women utilize test results to guide treatment. For example, NCCN guidelines suggest risk-reducing mastectomy and BSO should be considered for women with *BRCA1* and *BRCA2* mutations (12). In our study, of the six women with clinically-detected PV in *BRCA2*, three underwent BM and BSO at the time of diagnosis and remain cancer free. The remaining three women with PVs in *BRCA2* developed ipsilateral breast cancer with one woman having ovarian cancer found incidentally during her subsequent BSO. Each of these three women was test-eligible at the time of DCIS diagnosis and could have potentially prevented additional cancers had they pursued timely genetic testing and risk-reducing surgeries.

While the benefit of risk-reducing surgeries in preventing second cancers in women with DCIS and PV in *BRCA1* or *BRCA2* are well established, preventing recurrence without overtreatment for women with PV in other genes is more challenging. This is of considerable importance as within our cohort, 31/39 PV were in genes other than *BRCA1* and *BRCA2*. Of note, nine (1.9%) of women had PV in *CHEK2* of which two had second cancer events. Currently, enhanced surveillance rather than risk-reducing surgery is recommended for women with *CHEK2* PV (12). In conjunction with our results, Petridis et al. found that 5/16 (31%) of women with DCIS and *CHEK2* mutations developed contralateral disease (13) and a recent study of germline variants in patients with second breast cancers found that *CHEK2* was the most frequently mutated gene in women with second breast cancers (3.4%) (14). Thus, the risk of recurrence for women with DCIS and *CHEK2* PV may warrant the use of risk-reducing surgery.

In conjunction with the high frequency of PV in *CHEK2*, *MUTYH* (n=10, 2.1%) was the gene with the highest frequency of PV in our study, with two of the 10 women recurring. While a study of over 30,000 women with breast cancer found no significant increase in risk of breast cancer in women with *MUTYH* (15), a recent study of 165 women with *BRCA1/2*-negative IBC found that *MUTYH* was the most commonly mutated gene (3.6%) (16). The use of expanded germline panel testing may add complexity to patients understanding of DCIS and surgical decision making.

There are several limitations to this study. Data were not available for pre- or post-test counseling, thus, it is not possible to determine how many patients did not undergo genetic testing because they were not offered the opportunity and how many declined testing. In addition, the reasons behind surgical decision making were not collected, thus, we were unable to determine why in women who had testing before definitive surgery, 42.9% of women who received negative test results elected to undergo BM and 40% with a *BRCA2* PV chose BCS. Rates of contralateral breast cancer are low in women undergoing BCS, with use of radiation associated with lower risk for ipsilateral breast cancer and endocrine therapy associated with risk of contralateral breast cancer (17). BM may, therefore, represent overtreatment for the majority of patients with DCIS. Genetic testing to identify those women with PV at increased risk for SCE provides important information that will allow the patient to develop an individualized and tailored breast cancer treatment plan. Surgical decision should balance the risks and morbidity associated with BM with desired cosmesis, extent of DCIS, concerns about side effects of radiation therapy and fear of recurrence. Finally, although germline status was available for 484 women in this study, the number of women with PV was small. Thus, care must be taken in interpreting risk of SCE or using these data to influence surgical decision making.

In conclusion, 8.1% of women with unilateral DCIS had detectable germline mutations, including 3.4% of women not currently eligible for genetic testing. Less than half of the eligible women pursued genetic testing, and 10% did so only after a second cancer event. Half of the women with PV in genes for which prophylactic surgery should be discussed did not undergo BM and recurred. Given that more than 50% of PV were detected only in the research setting and that SCE were significantly higher in women with PV compared to those without, we suggest that, in accordance with recommendations from the ASBS, germline testing should be offered to all women diagnosed with DCIS, and in fact, should be included as standard-of-care at the time of diagnosis. Future studies to identify the factors associated with the suboptimal pursuit of genetic testing and subsequent risk-reducing surgeries are critical to reduce the risk of second cancer events in women with DCIS.

## Data availability statement

The original contributions presented in the study are included in the article/supplementary materials. Further inquiries can be directed to the corresponding author.

## Ethics statement

The studies involving human participants were reviewed and approved by Walter Reed National Military Medical Center Human Use Committee and Institutional Review Board. The patients/participants provided their written informed consent to participate in this study.

## Author contributions

LT reviewed the data and provided extensive revisions to the manuscript; LL generated panel test sequencing data and contributed to revisions of the manuscript; CT provided clinical interpretation of the data and revision of the manuscript, CS provided resources for the study and revision of the manuscript, RE conceived of the project, performed data analysis and wrote the manuscript. All authors contributed to the article and approved the submitted version.

## Funding

This research was supported by a cooperative agreement from the Uniformed Services University of the Health Sciences HU0001-16-2-0004 through the Henry M. Jackson Foundation for the Advancement of Military Medicine, Inc.

## Acknowledgments

The contents of this publication are the sole responsibility of the author(s) and do not necessarily reflect the views, opinions or policies of the Uniformed Services University of the Health Sciences (USUHS), the Henry M. Jackson Foundation for the Advancement of Military Medicine, Inc., the Department of Defense (DoD) or the Departments of the Army, Navy, or Air Force. Mention of trade names, commercial products, or organizations does not imply endorsement by the U.S. Government.

## Conflict of interest

The authors declare that the research was conducted in the absence of any commercial or financial relationships that could be construed as a potential conflict of interest.

## Publisher’s note

All claims expressed in this article are solely those of the authors and do not necessarily represent those of their affiliated organizations, or those of the publisher, the editors and the reviewers. Any product that may be evaluated in this article, or claim that may be made by its manufacturer, is not guaranteed or endorsed by the publisher.
